# Retrograde Transport of Tobacco Phytaspase Is Mediated by Its Partner, Tubby-like F-Box Protein 8

**DOI:** 10.3390/ijms26052236

**Published:** 2025-03-02

**Authors:** Raisa A. Galiullina, Artemii A. Pigidanov, Grigoriy G. Safronov, Svetlana V. Trusova, Anastasia D. Teplova, Sergei A. Golyshev, Marina V. Serebryakova, Irina E. Kovaleva, Anastasia V. Litvinova, Nina V. Chichkova, Andrey B. Vartapetian

**Affiliations:** 1 Belozersky Institute of Physico-Chemical Biology, Lomonosov Moscow State University, Moscow 119991, Russia; raisa-galiullina@rambler.ru (R.A.G.); svetlana.fedoseeva@gmail.com (S.V.T.); sergei.a.golyshev@gmail.com (S.A.G.); mserebr@mail.ru (M.V.S.); kovaleva@belozersky.msu.ru (I.E.K.); chic@belozersky.msu.ru (N.V.C.); 2 Faculty of Bioengineering and Bioinformatics, Lomonosov Moscow State University, Moscow 119991, Russia; artemiy.pigidanov@mail.ru (A.A.P.); grigorij.safronov@gmail.com (G.G.S.); anastasia_teplova@mail.ru (A.D.T.); nasta.litwinova@gmail.com (A.V.L.)

**Keywords:** endocytosis, oxidative stress, phytaspase, plant cell, subtilisin-like protease, protein interaction, protein trafficking, Tubby-like F-box protein

## Abstract

Phytaspases, plant cell death-promoting and proprotein-processing proteolytic enzymes of the plant subtilase family, display aspartate (caspase-like) cleavage specificity and a very unusual retrograde trafficking from the apoplast to the cell interior upon induction of death-inducing stresses. To determine the underlying molecular mechanisms, we performed a search for tobacco phytaspase (*Nt*Phyt) interactors using an in vivo cross-linking approach in *Nicotiana tabacum* plants. Tobacco Tubby-like F-box protein 8 (named Tubic hereafter) was identified as an *Nt*Phyt interactor, with formation of the cross-linked complex being only efficient under the oxidative stress conditions. Direct interaction of the two proteins was further corroborated in the in vitro experiments. Analysis of Tubic-EGFP behavior in plant cells revealed that Tubic is a membrane-associated and fairly unstable protein. Furthermore, we showed that *Nt*Phyt and Tubic are capable of negatively affecting one another in plant cells. On the other hand, down-regulation of Tubic in Tubic-silenced plants impaired specifically the retrograde transport of *Nt*Phyt upon the induction of oxidative stress, testifying to a critical role of Tubic in this process. Our study, thus, contributes to understanding of the mechanisms of *Nt*Phyt retrograde trafficking in plant cells subjected to stress.

## 1. Introduction

In living cells, the delivery of proteins to the right place at the right time is essential to support cell functioning and organismal homeostasis. This holds true for phytaspases, a cell death-related group of plant subtilisin-like proteases (subtilases) possessing a rarely occurring aspartate cleavage specificity (hence, their name) [1,2,3,4], resembling that of animal apoptotic proteases, caspases [5,6]. Under normal conditions, phytaspases, like the majority of plant subtilases, are secreted from the cell [1,7]. In the course of this anterograde journey, phytaspases become autocatalytically processed through the detachment of the inhibitory prodomain of their precursor protein, and the mature proteolytically active enzyme is eventually released into the apoplast [1]. However, under death-inducing conditions, such as the hypersensitive response to the tobacco mosaic virus infection, or oxidative stress, active phytaspases are translocated from the apoplast back toward the cell interior [1,8] with the aid of clathrin-mediated endocytosis [9,10]. Up-regulation of the phytaspase level stimulates plant cell death, whereas down-regulation of the phytaspase activity confers enhanced resistance towards cell death-inducing insults [1,11,12]. Also consistent with a pro-death role of the stress-internalized enzyme, the inhibition of clathrin-mediated endocytosis was shown to prevent both retrograde transport of phytaspase and cell death [10].

Within the trafficking route of phytaspases, retrograde transport of the enzyme is the most unusual and mechanistically, the least understood step. In an attempt to fill this gap, here, using an in vivo chemical cross-linking approach, we undertook a search for phytaspase interactors that might contribute to the stress-induced internalization of *Nicotiana tabacum* phytaspase (*Nt*Phyt). As a result, we identified tobacco Tubby-like F-box protein 8 as an *Nt*Phyt partner/interactor.

The vertebrate family of Tubby-like proteins (TULPs) includes the founding member Tubby and related TULPs, TULP1 to TULP4 [13]. Mutations in mouse Tubby lead to maturity-onset obesity, blindness, and deafness [14,15]. A distinctive feature of this protein family is the presence of a Tubby domain (~260 amino acid residues long) at the C-terminus. This domain comprises a closed β barrel consisting of 12 antiparallel strands, surrounding a central hydrophobic α helix [16,17]. Specific binding of the Tubby domain to phosphoinositides, in particular to the most abundant phosphoinositide of the inner leaflet of the plasma membrane phosphatidylinositol 4,5-bisphosphate (PI(4,5)P_2_), was demonstrated [17]. Two motifs within the Tubby domain are responsible for this interaction, both containing essential basic amino acid residues [17,18]. The association of the Tubby domain with the plasma membrane appears to be regulated. Activation of phospholipase C-β (PLC-β) through Gα_q_-coupled GPCRs (G-protein-coupled receptors) reducing the membrane PI(4,5)P_2_ level was shown to induce dislodgment of Tubby and other TULPs from the plasma membrane and their subsequent nuclear translocation. Likewise, mutations in the conserved PI(4,5)P_2_ -binding residues of TULPs resulted in their translocation to the nucleus [17].

The amino terminus of TULPs is varied and in several cases (Tubby, TULP1 and TULP3) binds to the intraflagellar transport complex, IFT-A [19]. Consistently, Tubby and related TULPs were implicated in the trafficking of cargo proteins including GPCRs from PI(4,5)P_2_-rich plasma membrane domains into primary cilia that are deficient in PI(4,5)P_2_ [19]. Other studies implicated TULPs in endocytic vesicle trafficking as well. For instance, Tubby was shown to be involved in an endocytic pathway regulating fat storage [20]. TULP1, a photoreceptor-specific protein, co-localizes with the heavy chain of clathrin, and its co-localization and interaction with dynamin-1 was reported [21,22]. Apart from the above findings related to intracellular functions of TULPs, a small proportion of Tubby and TULP1 (approx. 3%) was reported to be unconventionally secreted from P12 cells [23] and to facilitate retinal pigment epithelium and macrophage phagocytosis [24].

Plants also have an even broader family of Tubby-like proteins (commonly abbreviated as TLPs), e.g., encompassing 11 members in *Arabidopsis thaliana* [25,26], many of which are expressed ubiquitously. The Tubby domain of at least some plant TLPs appears to share the PI(4,5)P_2_- binding capacity with their animal counterparts, and the majority of the *A. thaliana* TLPs are targeted to the plasma membrane [27,28]. Notably, treatment of *A. thaliana* leaf cells with 0.3 M NaCl, 0.4 M mannitol, or 20 mM H_2_O_2_ caused translocation of the Tubby domain of *At*TLP3 from the plasma membrane into the cytosol and nucleoplasm in a phospholipase C-dependent manner [27].

A distinctive feature of plant TLPs is the almost ubiquitous presence of an F-box domain as an N-terminal functional domain that precedes the Tubby domain. The presence of an F-box domain implies a link between plant TLPs and ubiquitin ligases. SKP1-Cullin-F-box (SCF)-type E3 ubiquitin ligase complexes use a family of F-box proteins as substrate adaptors to mediate the degradation of a large number of regulatory proteins [29,30,31]. Indeed, the interaction of the F-box domain in many plant TLPs with SKP1 proteins was substantiated [25,28]. Furthermore, *A. thaliana* TLP6 was shown recently to target phosphoinositide biosynthesis-related kinases for ubiquitination and degradation [32].

For a number of *A. thaliana* TLPs, involvement in abscisic acid (ABA) signaling has been documented [25,28]. *At*TLP9 mutants are ABA-insensitive, whereas the *At*TLP9 overexpressors were hypersensitive to ABA [25]. Available data suggest a role of plant TLPs in stress responses, as overproduction of TLPs frequently enhances stress tolerance and positively regulates immune responses [27,32,33,34,35]. However, the underlying molecular mechanisms are largely unknown.

Here, in continuation of our study and using *Nt*Phyt as the newly identified TLP interactor, we provide evidence that *N. tabacum* Tubby-like F-box protein 8 mediates retrograde translocation of *Nt*Phyt under stress-inducing conditions.

## 2. Results

### 2.1. Identification of a Phytaspase Interactor Using Chemical Cross-Linking in N. tabacum Leaves

**Figure 1:**
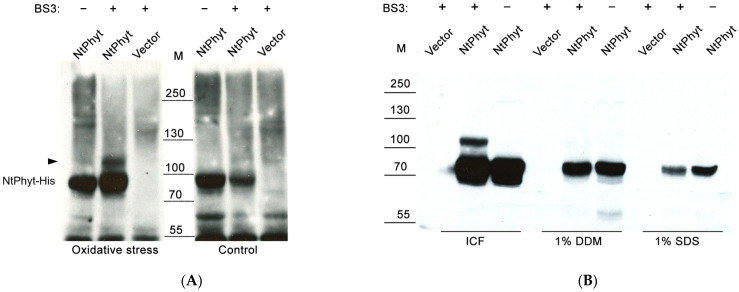
Looking for a phytaspase interactor using chemical cross-linking. *N. tabacum* leaves transiently producing *Nt*Phyt-His (*Nt*Phyt) or infiltrated with agrobacteria bearing the empty vector (vector) were cut into pieces, infiltrated either with 20 µM antimycin A (oxidative stress samples) or with water (control), and kept for 4 h in the dark. Extracellular liquid was then removed using low-speed centrifugation, and the samples were infiltrated with 2 µM BS3 cross-linking reagent (BS3+) or with buffer only (BS3−). After incubation for 30 min, the cross-linking reaction was stopped via infiltration with Tris buffer. (**A**) Water-soluble proteins extracted from equal weight amounts of the leaf samples were separated with SDS 6–12% gradient polyacrylamide gel electrophoresis (PAGE) and examined using Western blotting (WB) with HisProbe-HRP detection. The position of *Nt*Phyt-His (~80 kDa) is indicated. The arrowhead points to a ~110 kDa protein band in the *Nt*Phyt-His-producing sample treated with antimycin A and BS3, possibly representing a cross-linked complex. M, positions of the MW protein markers. (**B**) Proteins from the *Nt*Phyt-His-producing leaves treated with antimycin A and BS3 were fractionated to obtain intracellular soluble proteins (IFC), the residual leaf material was re-extracted with dodecyl maltoside (1% DDM) and 1% SDS to obtain the membrane protein fractions. Proteins from all three fractions were purified using Ni-NTA affinity chromatography and analyzed using WB using HisProbe-HRP detection. Note that the intensities of the free (non-cross-linked) *Nt*Phyt-His bands may serve as a loading control.

To perform a search for proteins interacting in vivo with *Nt*Phyt, we transiently produced *Nt*Phyt bearing a C-terminal His_6_-tag in *N. tabacum* leaves by means of agroinfiltration. This minor modification of the enzyme was shown previously not to interfere with the *Nt*Phyt proteolytic activity and localization [36], yet it allowed us to visualize *Nt*Phyt and its putative complexes on Western blots and to purify them from leaf extracts. Control leaves were infiltrated with agrobacteria bearing the empty vector. Three days post agroinfiltration (dpi), the leaf pieces were vacuum-infiltrated with 20 µM antimycin A to induce oxidative stress, *Nt*Phyt internalization, and cell death, or with water (control). Upon 4 h of incubation, the extracellular liquid was removed, and the leaf pieces were infiltrated with a water-soluble and membrane-impermeable bifunctional cross-linking reagent BS3 (bissulfosuccinimidyl suberate, which has a spacer arm of 11 Å) for 30 min. A separate group of leaf pieces received a buffer only. After stopping the cross-linking reaction, the soluble protein fractions from all types of samples were analyzed using Western blotting for the presence of cross-linked *Nt*Phyt derivatives, that is, phytaspase-containing (His tag-positive) bands with an electrophoretic mobility slower than that of the intact *Nt*Phyt-His (~80 kDa). Figure 1A demonstrates that such a band (approx. 110 kDa) was indeed observed in the *Nt*Phyt-His-producing sample that was subjected to oxidative stress and cross-linking treatment. Of note, this band was absent in the *Nt*Phyt-His-producing sample that was not treated with BS3, and in the BS3 cross-linked control sample without the *Nt*Phyt-His production (Figure 1A). Therefore, we concluded that this 110 kDa band likely represents a complex of *Nt*Phyt-His with a cross-linked protein. Importantly, this band was not detected in the BS3 cross-linked *Nt*Phyt-His—producing leaf sample that was not subjected to the oxidative stress treatment (Figure 1A), thus, raising the possibility that the formation of the complex may be associated with the induction of *Nt*Phyt-His internalization.

To further address the origin and content of the putative *Nt*Phyt-containing complex, several subcellular protein fractions (i.e., intracellular soluble proteins (ICF), membrane proteins extractable with dodecyl maltoside (DDM) and sodium dodecylsulfate (SDS) detergents) were obtained from *Nt*Phyt-His-producing leaves treated with antimycin A and BS3 cross-linker. Unrelated proteins were eliminated from these fractions by means of Ni-NTA affinity chromatography, and the *Nt*Phyt-His-containing eluates were analyzed using Western blotting with HisProbe-HRP detection. As shown in Figure 1B, the 110 kDa band corresponding to the putative cross-linked complex was clearly visible in the fraction of intracellular soluble proteins, while it was undetectable in the membrane protein fractions (Figure 1B). As previously mentioned, the formation of this band depended on the *Nt*Phyt-His production and on the BS3 treatment (Figure 1B).

**Figure 2:**
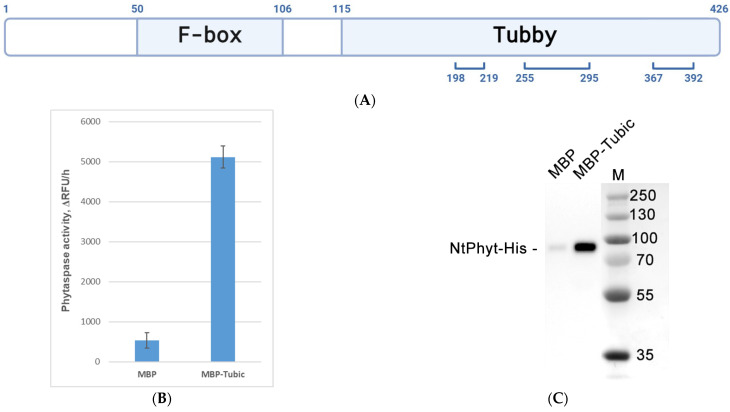

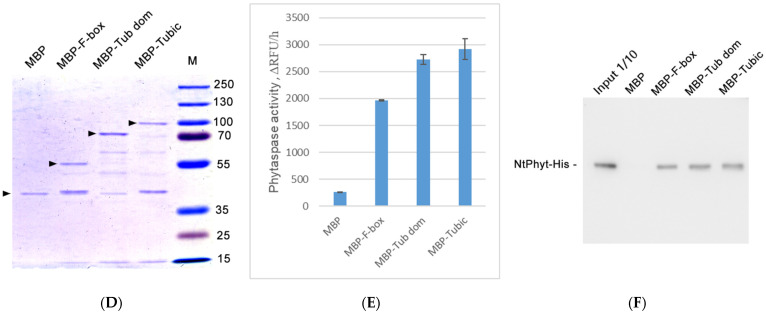
Evidence for the direct in vitro interaction of *Nt*Phyt with Tubic. (**A**) Schematic representation of Tubic. Boundaries of the F-box and Tubby domains are indicated above the structure. Bottom brackets show positions of tryptic peptides of Tubic identified by MS analysis. (**B**,**C**) Equivalent amounts of the Amylose resin-immobilized MBP-Tubic and MBP were incubated with *Nt*Phyt-His. After the elution of the resin-bound proteins with maltose-containing buffer, *Nt*Phyt proteolytic activity was fluorimetrically determined in the eluates using 20 µM Ac-VEID-AFC as a phytaspase substrate (**B**). Relative rates of hydrolysis were determined as an increase in relative fluorescence units per hour (∆RFU/h). In parallel, the eluates were analyzed using WB with HisProbe-HRP detection for *Nt*Phyt-His visualization (**C**). Position of *Nt*Phyt-His is indicated. M, molecular weight markers. (**D**) Tubic derivatives (full-length Tubic, F-box domain, Tubby domain (Tub dom)) fused to MBP were overproduced in *E. coli* cells and purified using Amylose resin affinity chromatography. Eluates from the resin were analyzed using SDS gel electrophoresis in a 12% gel with Coomassie blue staining. Arrowheads indicate the positions of intact proteins. (**E**,**F**) Both of the functional domains of Tubic are capable of *Nt*Phyt binding. Assessment of *Nt*Phyt-His interaction with the amylose resin-immobilized MBP-F-box domain and MBP-Tubby domain (Tub dom) was performed as described in (**B**,**C**). Free MBP and MBP-Tubic samples were included as negative and positive controls, respectively. The amounts of the affinity resin-bound *Nt*Phyt-His were analyzed in the eluates from the resin by measuring *Nt*Phyt-His proteolytic activity (**E**) and using WB with HisProbe-HRP detection (**F**). Input 1/10 indicates one-tenth of the amount of *Nt*Phyt-His taken to perform an in vitro binding assay. In (**B**,**E**), data represent the mean ± SD of three independent experiments.

To identify the components of the putative 110 kDa protein complex, protein content from the excised band was characterized using an MALDI-TOF analysis of tryptic peptides. Alongside with peptides originating from *Nt*Phyt-His, a set of peptides corresponding to the predicted *N. tabacum* Tubby-like F-box protein 8 (Protein ID A0A1S4C7Y4, MW ~47 kDa) was identified (Figure 2A). In plants, proteins of this family usually contain two characteristic domains: a Tubby-like domain at the C-terminus and an F-box domain close to the N-terminus of the protein. For the sake of brevity, we named this newly discovered protein ‘Tubic’.

### 2.2. Evidence for Direct NtPhyt-Tubic Interaction Using Purified Proteins

To verify the results of our in vivo cross-linking approach, we tested whether the interaction between *Nt*Phyt and Tubic is direct and could be demonstrated with purified components in vitro. To this end, the *N. tabacum* Tubic gene was cloned and the protein was overproduced in *E. coli* cells in the form of an MBP (maltose binding protein)-Tubic fusion protein. The protein was isolated using affinity chromatography on Amylose resin, and the interaction of the immobilized fusion protein with *Nt*Phyt-His was assessed. Proteolytically active *Nt*Phyt-His for this assay was transiently overproduced in *N. benthamiana* leaves and isolated from the apoplast with the aid of Ni-NTA affinity chromatography. Quantification of the phytaspase proteolytic activity eluted with maltose upon incubation of *Nt*Phyt-His with the immobilized MBP-Tubic fusion protein demonstrated that the formation of the *Nt*Phyt-Tubic complex could be readily achieved with the purified proteins—Figure 2B. Likewise, analysis of the eluates using Western blotting with HisProbe-HRP detection confirmed the specific binding of *Nt*Phyt to MBP-Tubic, while its interaction with free MBP was negligible (Figure 2C).

To learn which of the functional domains of Tubic may be responsible for *Nt*Phyt binding, MBP was fused either to the N-terminal portion of Tubic encompassing the F-box domain (Tubic amino acid residues 1–115) or to the Tubby domain (Tubic residues 116–426). The recombinant proteins were isolated from *E. coli* cells (Figure 2D), immobilized on the Amylose resin, and tested for *Nt*Phyt-His binding as above. Surprisingly, we were able to observe comparably efficient *Nt*Phyt-His interaction with both Tubic derivatives (but not with free MBP), judging by the determination of the phytaspase proteolytic activity in the maltose eluates from the respective column (Figure 2E) and using a Western blot analysis of the eluates with HisProbe-HRP detection (Figure 2F).

Collectively, these data indicate that *Nt*Phyt can bind Tubic both in vivo and in vitro, that the interaction between the two proteins is direct, and that each functional domain of Tubic is capable of *Nt*Phyt binding.

### 2.3. Tubic Behavior in Plant Cells

**Figure 3:**
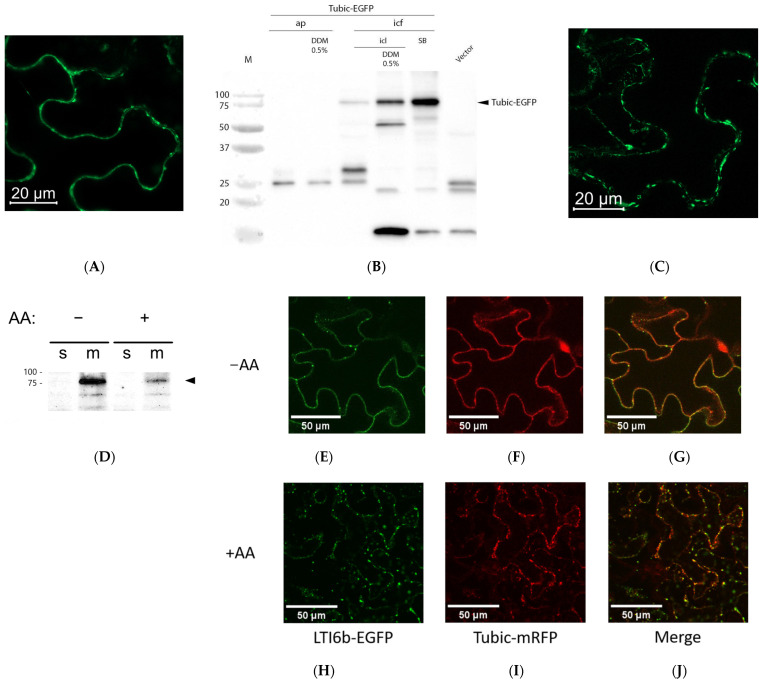
Localization of Tubic in *N. benthamiana* cells. (**A**) Tubic-EGFP was transiently produced in *N. benthamiana* leaves using agroinfiltration and examined using confocal fluorescence microscopy at 2 dpi. (**B**) Subcellular fractionation of proteins from Tubic-EGFP-producing leaves. The following protein fractions were obtained in sequential order: soluble apoplastic proteins (ap); proteins from the apoplastic wash with 0.5% DDM-containing buffer (ap + 0.5% DDM); intracellular fractions (icf) included: intracellular soluble proteins extractable with ICL buffer (icl); proteins solubilized in the presence of 0.5% DDM (icl + 0.5% DDM); water-insoluble membrane protein fraction (SB) obtained by boiling the residual leaf material in Sample Buffer. Total protein fraction obtained from leaves infiltrated with agrobacteria carrying the empty vector served as a control (vector). Proteins were separated with 12% SDS-PAGE and analyzed using WB with an anti-EGFP antibody. The arrowhead indicates the position of Tubic-EGFP (MW ~80 kDa). M, molecular weight markers. (**C**) Confocal fluorescence microscopy localization of Tubic-EGFP in leaves treated with 50 µM antimycin A for 9 h. The brightness of the image was increased five-fold (relative to panel (**A**)) to allow clear visualization. (**D**) The membrane-binding capacity of Tubic-EGFP is not perturbed in the antimycin A-treated leaves. The soluble protein (s) and membrane protein (m) fractions from leaf pieces either non-treated (−AA) or treated with antimycin A (+AA) were separated with 10% SDS PAGE. WB visualization was performed using an anti-EGFP antibody. The arrowhead indicates the position of Tubic-EGFP. (**E**–**J**) Confocal fluorescence microscopy visualization of LTI6b-EGFP (**E**,**H**) and Tubic-mRFP (**F**,**I**) co-produced in *N. benthamiana* leaves via agroinfiltration. The upper row: non-stressed leaves (−AA); the bottom row, leaves treated overnight with 15 µM antimycin A (+AA). (**G**,**J**), merged images demonstrating partial co-localization of the two proteins.

To characterize the behavior of Tubic in a plant cell, Tubic-EGFP protein was produced in *N. benthamiana* leaves by means of agroinfiltration. Confocal fluorescence microscopy examination revealed localization of Tubic-EGFP at the periphery of epidermal cells (Figure 3A). As the Tubby domain is known to associate with the cytoplasmic side of the cell membrane through binding to phosphoinositides, phosphoinositide (4,5) bisphosphate in particular [17], we asked whether tobacco Tubic could behave similarly. The separation of leaf tissues containing Tubic-EGFP into the apoplastic, intracellular soluble, and detergent-soluble protein fractions with subsequent Western blot analysis revealed the predominant presence of Tubic-EGFP in the membrane-containing (detergent-soluble) protein fraction, a low yet clearly detectable level of Tubic-EGFP in the intracellular soluble protein fraction, and its absence in the apoplastic wash (Figure 3B).

To learn whether stress conditions could affect the intracellular localization of Tubic-EGFP, Tubic-EGFP-producing leaf tissues were subjected to oxidative stress via treatment with 50 µM antimycin A. Confocal fluorescence microscopy analysis of these tissues revealed a significant weakening of the Tubic-EGFP signal, and the presence of Tubic-EGFP in punctate structures (Figure 3C), suggesting that some redistribution of Tubic-EGFP has occurred in response to stress. However, the membrane-binding capacity of Tubic-EGFP was still retained, as inferred from the results of protein fractionation analysis (Figure 3D).

We then compared the localization of Tubic-mRFP with that of the LTI6b-EGFP protein, a characteristic plasma membrane marker [37]. Being a component of the cell membrane, LTI6b is subjected to clathrin-mediated endocytosis (CME), with the subsequent recycling back to the plasma membrane [38]. Under normal conditions, the co-production of Tubic-mRFP and LTI6b-EGFP resulted in a peripheral localization of both proteins (Figure 3E–G), consistent with their membrane-binding properties. Upon the induction of oxidative stress via leaf treatment with antimycin A, the localization of both proteins changed from ‘smooth peripheral’ to ‘punctate’, with the fluorescent dots being located both close to the cell border and inside the cell—Figure 3H–J. For LTI6b, this might be indicative of enhanced endocytosis or impaired recycling. Interestingly, partial co-localization of Tubic with LTI6b was observed under stress conditions (Figure 3J), suggesting that the pathways of the two proteins inside the cell might overlap.

### 2.4. Instability of Tubic

**Figure 4:**
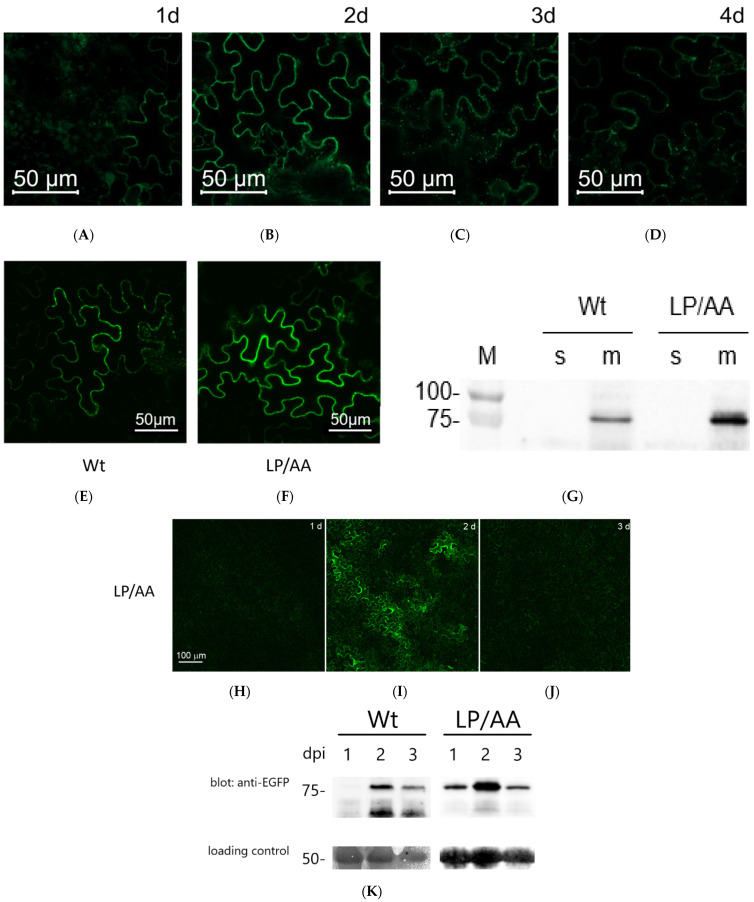
Stability evaluation of Tubic and of the LP/AA Tubic mutant. (**A**–**D**) Confocal fluorescence microscopy of *N. benthamiana* leaves transiently producing Tubic-EGFP at various dpi. Identical microscope settings were used to obtain the images. (**E**–**G**) Comparison of relative levels of Tubic-EGFP and LP/AA Tubic-EGFP in *N. benthamiana* leaf cells at 2 dpi using confocal fluorescence microscopy examination (**E**,**F**) and WB analysis of the soluble (s) and membrane-bound (m) protein fractions (**G**). Anti-EGFP antibody was used for the on-blot visualization. (**H**–**J**) Relative levels of the LP/AA Tubic-EGFP mutant in plant cells at 1 to 3 dpi were assessed using confocal fluorescence microscopy. (**K**) WB analysis with anti-EGFP antibody of the relative levels of wild-type Tubic-EGFP and LP/AA Tubic-EGFP at different dpi. The lower panels in (**K**): the loading control depicting the Ponceau S-stained Rubisco band.

In the course of the above studies, we noticed that the ectopically produced Tubic fails to accumulate to a high level in plant cells. First, the Tubic signal on Western blots was rather weak (requiring long exposure time), despite the fact that the Tubic gene was placed under the control of a strong and constitutively active 35S promoter. Second, confocal fluorescence microscopy inspection of Tubic-EGFP-producing leaves at different days post infiltration (dpi) revealed that the Tubic-EGFP signal, peaking at 2 dpi, becomes significantly weaker at later time points (Figure 4A–D). In contrast to this behavior, free EGFP demonstrated constant accumulation during the same period (Supplementary Figure S1).

We reasoned that the apparent instability of Tubic might be due to the presence of an F-box in its structure (see Figure 2A). Proteins possessing such a domain commonly serve as the substrate recognition subunit of SKP1-Cullin-F-box (SCF) ubiquitin ligases through their binding to E3 ligase constituents, e.g., SKP1 protein, and may also be controlled through autoubiquitination mediated by the SCF scaffold [39]. To test this hypothesis, two amino acid residues of the Tubic F-box (Leu^56^Pro^57^) corresponding to those engaged in contact with SKP1 [40] were mutated (the LP/AA mutant). Fluorescence microscopy examination and Western blot comparison of the levels of the wild-type and mutant Tubic-EGFP accumulating in *N. benthamiana* leaf cells upon agroinfiltration showed marginal stabilization of the Tubic mutant versus the wild-type protein (Figure 4E–G), and the Tubic mutant retained the membrane association (Figure 4G). On the other hand, attenuation of the Tubic signal at later dpi was still evident even for the Tubic mutant (Figure 4H–K).

We conclude that although the putative interaction of Tubic with E3 ubiquitin ligase may affect the level of accumulation of Tubic, this is probably not the only factor that influences the stability of Tubic.

### 2.5. Phytaspase and Tubic: Antagonistic Relationships

**Figure 5:**
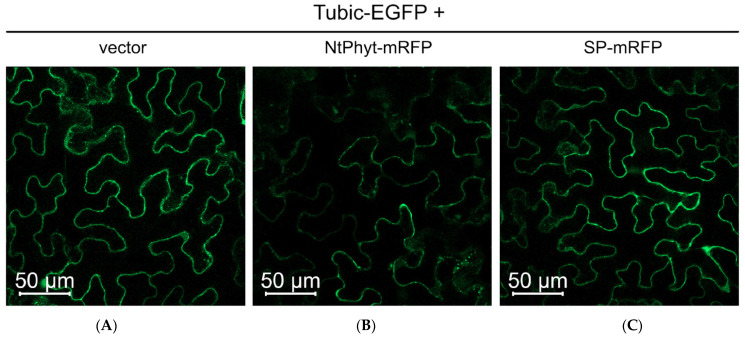

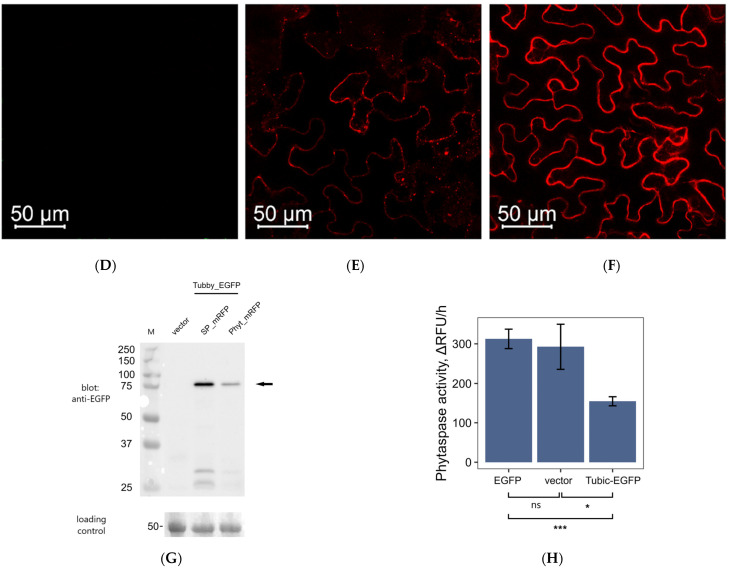
*Nt*Phyt and Tubic negatively affect one another. (**A**–**F**) Down-regulation of Tubic-EGFP in *N. benthamiana* cells co-producing *Nt*Phyt-mRFP (**B**), but not SP-mRFP (**C**), observed using confocal microscopy examination at 2 dpi. Vector, leaves were infiltrated with a mixture of agrobacteria carrying the Tubic-EGFP-encoding plasmid and the empty vector. The upper row (**A**–**C**)—green channel depicting the Tubic-EGFP fluorescence; the bottom row (**D**–**F**)—red channel depicting the *Nt*Phyt-mRFP or SP-mRFP fluorescence. Identical microscope settings were used to obtain the images within each row. (**G**) The total protein fractions from leaves co-producing Tubic-EGFP with either *Nt*Phyt-mRFP or SP-mRFP were obtained at 2 dpi and analyzed using WB with an anti-EGFP antibody. An extract from a leaf infiltrated with agrobacteria carrying the empty vector (vector) serves as a control. M, MW protein markers. The arrow points to the position of Tubic-EGFP. The lower panel in (**G**): the loading control. (**H**) Proteolytic activity of endogenous phytaspase in *N. benthamiana* leaves producing Tubic-EGFP or free EGFP, as compared to the vector only (vector) control. Phytaspase activity was analyzed in extracts using 20 µM Ac-VEID-AFC fluorogenic peptide substrate. Relative rates of hydrolysis were determined as an increase in relative fluorescence units per hour (∆RFU/h). Data represent the mean ± SD, *n* = 4. Single asterisk (*) represents *p* < 0.05, triple asterisk (***) represents *p* <  0.001 based on a *t* test with Benjamini-Hochberg correction; ns, not significant.

In an attempt to test the possibility of co-localization of Tubic with *Nt*Phyt, we co-produced Tubic-EGFP and *Nt*Phyt-mRFP in *N. benthamiana* leaves using agroinfiltration. Fluorescence microscopy inspection of the infiltrated leaves at 2 dpi, shown above to be the time point of maximum Tubic-EGFP accumulation, revealed, to our surprise, the presence of only low amounts of Tubic (Figure 5B). Control leaves producing Tubic-EGFP alone (without *Nt*Phyt-mRFP co-production) displayed clearly detectable fluorescence (Figure 5A), as expected. To verify whether this was the elevated level of *Nt*Phyt that caused Tubic down-regulation, several control experiments were performed. First, the co-production of SP-mRFP protein consisting of mRFP fused to the signal peptide (SP) of *Nt*Phyt failed to down-regulate the Tubic-EGFP level, as assessed using fluorescence microscopy and Western blot analyses (Figure 5C,G), although the mRFP protein (derived from SP-mRFP) accumulated in the apoplast of plant cells at a much higher level than *Nt*Phyt-mRFP (Figure 5E,F, red channel). Second, *Nt*Phyt-mRFP co-production did not cause weakening of the free EGFP fluorescence (Supplementary Figure S2), suggesting that *Nt*Phyt specifically affects the stability of Tubic. Furthermore, when the fluorescent protein tags were inverted (that is, when *Nt*Phyt-EGFP and Tubic-mRFP were co-produced), the *Nt*Phyt-dependent quenching of the Tubic fluorescence signal was still evident (Supplementary Figure S3).

We, thus, conclude that *Nt*Phyt is able to negatively affect the stability of Tubic.

To learn whether, in turn, Tubic could interfere with *Nt*Phyt, the specific proteolytic activity of endogenous *Nt*Phyt was quantitatively determined in extracts from leaves producing either Tubic-EGFP or free EGFP. As shown in Figure 5H, the production of Tubic-EGFP caused an approximately two-fold reduction of the level of *Nt*Phyt activity in leaf cells, whereas the effect of free EGFP, albeit overproduced to a much higher level than that of the fusion protein, was not significant.

It, thus, appears that the two interacting proteins, *Nt*Phyt and Tubic, are capable of negatively affecting one another.

**Figure 6:**
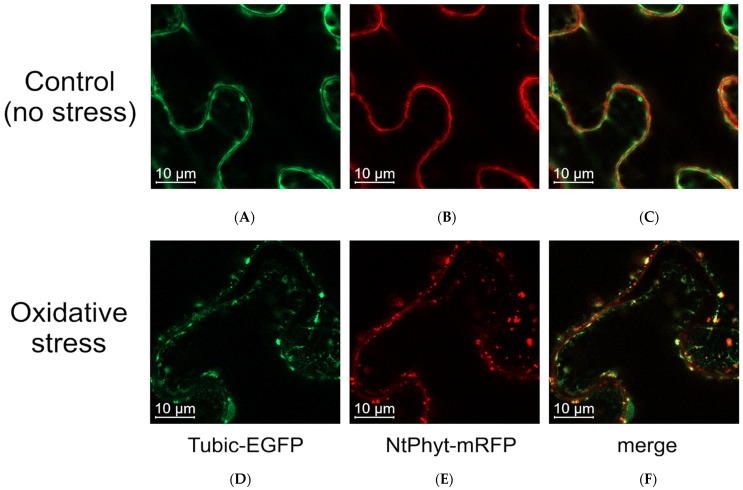
Confocal fluorescence microscopy visualization of Tubic-EGFP (**A**,**D**) and *Nt*Phyt-mRFP (**B**,**E**) co-produced in *N. benthamiana* leaves reveals differential localization of the proteins in the non-stressed tissue (**C**) and partial co-localization of the two proteins (**F**) upon the induction of oxidative stress (treatment with 50 µM antimycin A for 9 h).

Despite the observed attenuation of the Tubic-EGFP signal upon *Nt*Phyt production, confocal fluorescence microscopy still allowed us to perform co-localization studies of the two proteins. In non-stressed leaves co-producing Tubic-EGFP and *Nt*Phyt-mRFP, phytaspase was located within the apoplastic space, being surrounded by the Tubic-EGFP-labeled plasma membranes of neighboring cells (Figure 6A–C). Induction of oxidative stress in these leaves with antimycin A led to the formation of punctate structures, located both close to the cell borders and inside the cells (Figure 6D–F). Notably, partial co-localization of Tubic-EGFP with *Nt*Phyt-mRFP was observed in these structures (Figure 6F, merged, yellow signal).

We infer from this result that *Nt*Phyt and Tubic likely meet each other in the process of stress-induced *Nt*Phyt internalization.

### 2.6. Down-Regulation of Tubic Impairs Stress-Induced Retrograde Transport of NtPhyt

To get an idea of which processes in the *Nt*Phyt life and death cycle may require interaction with Tubic, the level of Tubic mRNA in *N. benthamiana* leaf cells was suppressed using the virus-induced gene silencing (VIGS) approach. To this end, Tobacco Rattle Virus (TRV) derivatives targeting two distinct regions of *N. benthamiana* Tubic mRNA were constructed, one of them being unique for Tubby-like F-box protein 1 of *N. benthamiana* displaying the highest similarity with *N. tabacum* Tubic, and another one shared by mRNA of *N. benthamiana* Tubby-like F-box protein 14. Approximately a two- to five-fold reduction of the Tubic mRNA level was achieved with both constructs in virus-infected leaves (Supplementary Figure S4), and similar ‘functional’ results were obtained upon *Nt*Phyt-mRFP production in the silenced leaves.

**Figure 7:**
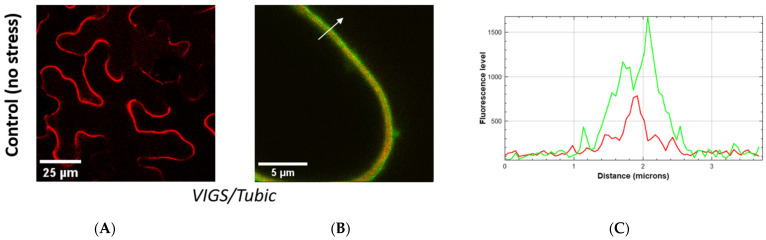

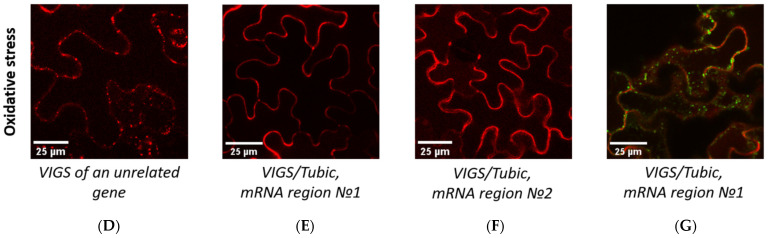
Down-regulation of Tubic via the VIGS approach does not impair *Nt*Phyt-mRFP secretion into the apoplast, but specifically precludes its retrograde transport upon stress induction. (**A**,**B**) *Nt*Phyt-mRFP and *Nt*Phyt-mRFP plus LTI6b-EGFP localization in the *Tubic*-silenced plants under normal conditions. (**C**) Measuring fluorescence intensities for *Nt*Phyt-mRFP (red) and LTI6b-EGFP (green) along the arrow (shown in (**B**)) confirms the apoplastic localization of *Nt*Phyt-mRFP. (**D**) Induction of oxidative stress via overnight leaf treatment with 20 µM antimycin A causes *Nt*Phyt-mRFP internalization in plants to be silenced for an unrelated gene (PDS). In the stressed leaves infected with the virus without any gene-specific insert, *Nt*Phyt-mRFP behaved similarly. (**E**,**F**) In the Tubic-silenced plants, however, *Nt*Phyt-mRFP preserves its smooth apoplastic localization despite the antimycin A treatment. (**G**) In the Tubic-silenced plants co-producing *Nt*Phyt-mRFP and LTI6b-EGFP, oxidative stress causes internalization of LTI6b-EGFP, but not of *Nt*Phyt-mRFP.

Suppression of the Tubic production appeared to exert no effect on the secretion of *Nt*Phyt-mRFP into the apoplast, with the protein being localized at the cell periphery (Figure 7A). More specifically, confocal fluorescence microscopy of the silenced leaves co-producing *Nt*Phyt-mRFP and the plasma membrane marker LTI6b-EGFP protein revealed *Nt*Phyt-mRFP (red) accumulation between the two LTI6b-EGFP-labeled (green) plasma membranes of neighboring cells (Figure 7B,C).

Upon the induction of oxidative stress via the antimycin A treatment of control leaves silenced for an unrelated gene (PDS, phytoene desaturase), retrograde transport of *Nt*Phyt-mRFP from the apoplast to inside the cell became visible, as indicative by the formation of fluorescent punctate structures both in the vicinity of the plasma membrane and inside the cell (Figure 7D). However, under the same conditions, *Nt*Phyt-mRFP in the Tubic-silenced plants preserved apoplastic localization (Figure 7E,F), indicating that its internalization was suppressed. As the stress-induced retrograde transport of *Nt*Phyt utilizes CME, this result might indicate that down-regulation of Tubic impairs CME, either in general or in a more *Nt*Phyt-specific way.

To distinguish between these possibilities, Tubic-silenced plants co-producing *Nt*Phyt-mRFP and LTI6b-EGFP were treated with antimycin A. Under these conditions, LTI6b-EGFP protein, whose plasma membrane localization depends on CME/recycling [38], became re-localized to inside the cell (green dots), suggesting that the CME-dependent uptake of plasma membrane-localized proteins was not generally abrogated (Figure 7G). However, *Nt*Phyt-mRFP preserved its apoplastic localization (Figure 7G), indicating that its uptake was specifically impaired.

We interpret these results to indicate that Tubic specifically mediates retrograde transport of *Nt*Phyt.

## 3. Discussion

One of the obvious approaches to deciphering the mode of functioning of an individual protein in the living cell is to look for its interactors. Here, we utilized this approach in relation to tobacco phytaspase, *Nt*Phyt, an exquisitely selective aspartate-specific protease of the subtilase family. From the functional point of view, *Nt*Phyt is known for its plant cell death-promoting activity [1,3,11], and is also involved in the specific processing of precursors of plant peptide hormones, prosystemin and prophytosulfokine [41,42]. A peculiar feature of *Nt*Phyt is its logistics. In healthy plant cells, the newly synthesized proteolytically active enzyme is secreted into the apoplast, where *Nt*Phyt is present in a soluble form [1]. However, a number of cell death-inducing conditions trigger retrograde transport of *Nt*Phyt to allow its access to intracellular components [1]. This unusual retrograde transport utilizes clathrin-mediated endocytosis [9,10] and does not require the proteolytic activity of *Nt*Phyt [43]. Notably, some kind of cell surface-localized receptor is likely required to allow internalization of the soluble apoplastic *Nt*Phyt.

By using an in vivo cross-linking approach, we identified Tubby-like F-box protein 8 of *N. tabacum* (here, named Tubic) as the in vivo *Nt*Phyt interactor. This conclusion was further supported by studying the interaction of the purified proteins in vitro.

In our study, efficient in vivo formation of the *Nt*Phyt-Tubic complex was observed upon induction of oxidative stress in *N. tabacum* leaf cells, that is, when retrograde transport of *Nt*Phyt was induced. This observation prompted the idea that interaction with Tubic may occur (and may be important) in the course of stress-induced internalization of *Nt*Phyt. The results obtained with Tubic-silenced plants which show that the down-regulation of Tubic specifically precludes stress-induced uptake of *Nt*Phyt are fully consistent with this assumption.

However, the interpretation of several phenomena observed in our study appears to be less straightforward. For example, Tubic lacks a signal peptide and is expected to be an intracellular protein associated with the inner face of the plasma membrane, while *Nt*Phyt accumulates in the apoplast. Then, how can these two proteins become cross-linked with BS3, a membrane-impermeable cross-linker? One possibility is that *Nt*Phyt was modified in the apoplast with one ‘arm’ of the bifunctional cross-linker, whereas the second ‘arm’ links Tubic in the process or after *Nt*Phyt translocation into the plant cell. There is yet another possibility. Low amounts of Tubby (less than 5%) were reported to be secreted in a non-canonical fashion out of mammalian cells [23]. In this setting, the interaction of Tubic with *Nt*Phyt could occur (and become fixed) in the apoplast. In our experiments with overproduced Tubic-EGFP, we failed to identify Tubic in the extracellular (apoplastic) fraction. Yet, very low amounts of the protein could escape detection and, thus, this possibility cannot be excluded completely.

Another thought-provoking observation is that ectopically produced Tubic-EGFP was predominantly found within the membrane-bound (detergent-soluble) protein fraction (Figure 3B). Yet the cross-linked *Nt*Phyt-Tubic complex was detected in the water-soluble protein fraction. To account for this discrepancy, we are considering the possibility that association with *Nt*Phyt could affect the membrane-binding properties of Tubic and lead to the release of the complex from the membrane.

The observation that *Nt*Phyt is able to interact both with the N-terminal fragment of Tubic encompassing the F-box domain and with the Tubby domain with comparable efficiency is also remarkable. Could the properties of the *Nt*Phyt-Tubic complex, such as targeting or stability, be distinct depending on whether the one or another binding site in Tubic (or both) is occupied with *Nt*Phyt? A perhaps related question is why are the relationships of the two partners apparently antagonistic and how could the cell make use of this behavior? Given that *Nt*Phyt is a death-promoting protease, and that Tubic is required for *Nt*Phyt retrograde transport, we hypothesize that perhaps this relationship could serve to neutralize unintended internalization of *Nt*Phyt. As soon as the intracellular level of *Nt*Phyt occasionally elevates under non-stressed conditions, Tubic becomes downregulated, and this in turn blocks further *Nt*Phyt uptake, thus, maintaining the low level of internalized *Nt*Phyt.

How Tubic might mediate the stress-induced internalization of *Nt*Phyt is an open question. Tubic apparently lacks a transmembrane domain(s) and, thus, by itself is unlikely to serve as a hypothetical *Nt*Phyt receptor. Data from animal cell studies indicate that TULPs frequently co-localize with the components of the CME machinery [21,22]. It, thus, can be envisaged that Tubic could be specifically involved in the endocytosis of a complex formed by *Nt*Phyt with its elusive receptor.

In conclusion, the observations that the plant Tubby-like protein Tubic is the *Nt*Phyt partner and is essential for the stress-induced internalization of the death-promoting protease reported here raise a number of important questions. These questions are worth addressing in future studies to obtain an understanding of the life and death decisions of the plant cell at the molecular level.

## 4. Materials and Methods

### 4.1. Plant Growth Conditions and Agroinfiltration

*N. tabacum* cv. Samsun and *Nicotiana benthamiana* plants were grown at 25 °C in soil in a controlled environment under a 16 h/8 h day/night cycle. For transient protein production, *Agrobacterium tumefaciens* C58C1 cells were transformed with the respective plasmid. Alternatively, cells double transformed with pLEX7000 and pBIB derivatives (see below), providing streptomycin plus spectinomycin resistance and kanamycin resistance, respectively, were obtained. To enhance the protein production in *N. benthamiana* plants, transformed cells were mixed with an equal number of agrobacteria bearing the p19 suppressor of silencing prior to infiltration. Agrobacteria carrying the empty vector were used as a control. Agrobacteria were infiltrated into leaves of 6-week-old plants using a blunt syringe. Typically, leaves were harvested 2 days post-infiltration (dpi), unless otherwise indicated in Figure legends.

### 4.2. Plasmid Construction

Construction of the *Nt*Phyt-His_6_, *Nt*Phyt-mRFP, *Nt*Phyt-EGFP, SP-mRFP, and SP-EGFP genes within the pLEX7000 expression vector [41] was described previously [1,10,36]. The EGFP-LTI6b-encoding plasmid (a pBIB-KAN vector derivative) was a gift from M. Taliansky (The James Hutton Institute, UK). cDNA encoding *N. tabacum* Tubby-like F-box protein 8 (Tubic, approx. 1300 bp long) was PCR-amplified using the primers Tubic_Bam_dir and Tubic_Sac_Xho_rev (see Supplementary Table S1 for primer sequences) and inserted downstream of and in frame with the MBP (maltose binding protein) gene between the *Nco*I and *Xho*I sites of the pET28a vector (Novagen, Madison, WI, USA) to generate the pET_MBP_Tubic plasmid. cDNAs encoding individual domains of Tubic were PCR-amplified using Tubic_Bam_dir plus F-box_z_Sac_rev (for the N-terminal domain) and Tub_dom_Bam_dir plus Tubic_Sac_Xho_rev (for the Tubby domain) primer pairs, respectively, and used to substitute the full-length Tubic gene in the pET_MBP_Tubic plasmid. To construct the control pET28-MBP plasmid, the MBP gene was amplified using the primers pET28_Sph_dir and MBP_z_Bam_rev and ligated between the *Sph*I and *Bam*HI sites of the pET28a vector.

For the in planta production of Tubic-EGFP and Tubic-mRFP, Tubic cDNA was amplified using the primers NtTubic_Nco_dir and NtTubic_Bam_rev and inserted upstream of and in frame with the EGFP or mRFP gene between the *Nco*I and *Sac*I sites of the pLEX7000 expression vector. The LP/AA Tubic mutant cDNA was obtained via megaprimer PCR using an internal mutagenizing primer NtTubic_LP/AA_ rev and external NtTubic_Nco_dir primer (for the 1st PCR step) and NtTubic_Bam_rev primer (for the 2nd step). Subsequently, the wild-type Tubic gene in the pLEX-Tubic_EGFP plasmid was substituted with the mutant one.

To construct TRV2 derivatives for the virus-induced gene silencing (VIGS), two cDNA fragments of *N. benthamiana* TLP1 were PCR-amplified using the NbTub1_VIGS_Kpn_dir plus NbTub1_VIGS_Bam_rev (a 148 bp long product) and NbTub1&14_VIGS_Xho_dir plus NbTub1&14_VIGS_Mlu_rev (a 349 bp long product) primer pairs. The DNA fragments were ligated between the corresponding sites of the pYL156 TRV2 vector [44,45] to produce TRV2:NbTLP1 and TRV2:NbTLP1&14 plasmids, respectively.

The identities of all constructs were confirmed using DNA sequence analysis. Bacterial and in-plant expression of the constructed protein-encoding genes was driven by the T7 promoter and dual cauliflower mosaic virus (*CaMV*) 35S promoter, respectively.

### 4.3. Transient Expression in Plants and Protein Fractionation

Leaves of *N. tabacum* and *N. benthamiana* plants were infiltrated with agrobacteria carrying the constructed binary plasmids in combinations described in Figure legends. At 2 dpi, leaf discs (approx. 15 mg each) from plants transiently producing the recombinant proteins were prepared and examined using confocal fluorescence microscopy. Where indicated, leaf discs were vacuum infiltrated with water containing 15 to 50 μM antimycin A (Sigma, St. Louis, MO, USA; from stock solution in ethanol) at the reduced pressure of 30 hPa (mbar) for 1 min and incubated for 4 h to overnight (depending on the concentration of antimycin A used) in the dark prior to microscopy examination. Control discs were infiltrated with distilled water supplemented with an equivalent amount of ethanol.

For protein fractionation, leaf pieces were vacuum infiltrated with MES100 buffer (20 mM MES, pH 5.5, and 100 mM NaCl) and centrifuged in a 1.5 mL centrifuge tube at 4 °C for 10 min at 2000× *g*. The apoplastic fluid was collected from the bottom of the tube. Where indicated, proteins from the extracellular space were re-extracted using vacuum infiltration with MES100 buffer supplemented with 0.5% DDM (n-dodecyl-β-D-maltopyranoside, dodecyl maltoside; Anatrace, Maumee, OH, USA) and centrifugation. The residual leaf material was frozen in liquid nitrogen and disrupted in Minilys homogenizer (Bertin Instruments, Montigny-le-Bretonneux, France) using 1.4 mm zirconium oxide beads for five 5 s bursts. An additional 10 s burst was performed after suspending the samples in ICL buffer (10 mM Tris-HCl, pH 8.8, 0.2 M NaCl, 30 mM magnesium chloride, 0.2 M sucrose, and 10 mM 2-mercaptoethanol), containing protease inhibitors aprotinin (2 µg/mL), leupeptin (6 µg/mL), chymostatin (6 µg/mL), E64 (6 µg/mL), 4-(2-aminoethyl)benzenesulfonyl fluoride (25 µg/mL), and 2 mM EDTA, and the sample was incubated on ice for 10 min. Water-insoluble material was pelleted using centrifugation for 10 min at 10,000× *g* at 4 °C, and the supernatant obtained represented the water-soluble fraction of the intracellular proteins.

Pellets obtained in the previous step were re-suspended in ICL buffer supplemented with 0.5–1% DDM and protease inhibitors via vortexing, incubated for 10 min on ice, and the detergent-solubilized protein fraction was obtained as a supernatant after centrifugation for 10 min at 10,000× *g* at 4 °C. The pellets, thus, formed (the membrane protein fraction) and were solubilized in Sample Buffer and boiled for 10 min prior to analysis via PAGE. To obtain ‘total protein’ samples, leaf pieces were disrupted in a homogenizer as described above and, after the addition of Sample Buffer, boiled for 10 min. Supernatants obtained after centrifugation for 10 min at 14,000× *g* were applied onto polyacrylamide gels.

### 4.4. In Vivo Cross-Linking

At 3 dpi, leaf pieces from *N. tabacum* plants either transiently producing *Nt*Phyt-His or infiltrated with agrobacteria carrying the empty vector were vacuum infiltrated with 20 μM antimycin A (to induce oxidative stress) or with water (non-stressed control) and incubated for 4 h in the dark. The apoplastic liquid was then removed via centrifugation at 2000× *g* for 10 min at 4 °C, and leaf pieces were infiltrated with 2 mM BS3 (bissulfosuccinimidyl suberate; Pierce, Rockford, IL, USA) cross-linking reagent dissolved in 0.1 M HEPES, pH 8.0 buffer, or with buffer only, and incubated for 30 min at room temperature. The excess reagent was then removed from the extracellular space by low-speed centrifugation, leaf pieces were additionally infiltrated with 0.1 M Tris-HCl, pH 8.0, buffer to stop the reaction and frozen.

Equal weight amounts of the leaf pieces were disrupted in Minilys homogenizer in the presence of ICL buffer (as described above) and analyzed using Western blotting with HisProbe-HRP detection. Alternatively, after separation of the soluble protein fraction via centrifugation at 14,000× *g* for 10 min at 4 °C, the pellet was extracted with the same buffer supplemented with 1% DDM, and then re-extracted with the buffer supplemented with 1% SDS to obtain fractions containing membrane proteins. Samples of all three fractions obtained, thus, were 10-fold diluted with 50 mM sodium phosphate buffer, pH 8.0, and separated via Ni-NTA agarose (Qiagen, Hilden, Germany) affinity chromatography using the resin (200 μL per sample) pre-washed with the same buffer. Flow-through fractions were discarded, the resin was washed with 6 × 0.7 mL of Wash buffer (50 mM sodium phosphate buffer, pH 8.0, 1 M NaCl, 10% glycerol), and the resin-bound proteins were eluted with B1 buffer (20 mM MES, 2 mM DTT, 0.1% DTT, and 5% glycerol), pH 5.5, containing 0.1 M EDTA (final pH 6.5). Samples were concentrated using Microcon-30kDa (Merck Millipore, Tullagreen, Ireland) and analyzed using Western blotting with HisProbe-HRP detection. The protein content of the 110 kDa phytaspase-containing band detected in the soluble protein fraction was characterized using mass spectrometry (MS) analysis.

### 4.5. In-Gel Trypsin Digestion and MALDI-MS Analysis

Pieces of about 5 mm^3^ of the stained protein-containing gel were destained three times with 20 mM NH_4_HCO_3_, pH 7.5, 40% aqueous acetonitrile solution. Reduction of cysteine residues with 10 mM dithiothreitol was carried out, and then alkylation with 30 mM iodoacetamide was performed. Gel pieces were dehydrated with 200 µL of 100% acetonitrile, and rehydrated with 5 µL of the digestion solution (pH 6) containing 15 µg/mL sequencing grade trypsin (Promega, Madison, WI, USA) in NH_4_HCO_3_, pH 7.5 aqueous solution. Digestion was carried out at 37 °C for 3 h. The resulting peptides were extracted with 5 µL of 0.5% TFA (trifluoroacetic acid), 30% acetonitrile solution. An aliquot of 1 μL of in-gel tryptic digest extract was mixed with 0.5 µL of 2,5-dihydroxybenzoic acid solution (40 mg/mL in 30% acetonitrile, 0.5% TFA). MALDI-TOF (matrix-assisted laser desorption ionization-time of flight) MS analysis was performed on an UltrafleXtreme MALDI-TOF-TOF mass spectrometer (Bruker Daltonics, Bremen, Germany). The MH+ molecular ions were measured in a reflector mode; the accuracy of monoisotopic mass peak measurement was within 50 ppm. Protein identification was carried out using MS ion search with the use of Mascot software version 2.3.02 (Matrix Science) through the home tobacco protein database. One missed cleavage, Met oxidation, Cys-propionamide or Cys-carbamidomethyl were permitted. Protein scores greater than 64 were considered to be significant (*p* < 0.05).

### 4.6. Production of Recombinant Proteins in Escherichia coli Cells and In Vitro Binding Assay

*E. coli* BL21 (DE3) cells were transformed with pET28a-based constructs encoding either MBP-Tubic, MBP-F-box domain, MBP-Tubby domain, or free MBP and grown in 40 mL of LB medium containing 0.2% glucose and 25 μg/mL kanamycin at 37 °C with shaking. Exponentially growing cells were shifted to 18 °C, induced with 0.5 mM isopropyl-β-D-thiogalactoside, and further incubated overnight at 18 °C with shaking. Cells were pelleted via centrifugation at 4000× *g* for 15 min at 4 °C, resuspended in 5 mL of CB buffer (20 mM Tris-HCl, pH 7.4, 200 mM NaCl, 1 mM DTT, 1 mM EDTA) containing protease inhibitors aprotinin (5 μg/mL), chymostatin (40 μg/mL), and 4-(2-aminoethyl)benzenesulfonyl fluoride (25 µg/mL), sonicated on ice for five 20 s bursts, and centrifuged at 14,000× *g* for 10 min at 4 °C. The cleared lysate (1.5 mL) was incubated with Amylose resin (New England Biolabs, Ipswich, MA, USA) (100 μL of an aqueous suspension per experiment, pre-washed with CB buffer) for 1 h at 4 °C with rotation. The resin was then washed with 4 × 1 mL of CB buffer, and stored on ice.

*Nt*Phyt-His was affinity-purified from the apoplast of *Nt*Phyt-overproducing *N. benthamiana* leaves using Ni-NTA agarose affinity chromatography as described in [36]. To assess Tubic-*Nt*Phyt binding, an aliquot (10 μL) of Amylose resin with immobilized MBP-Tubic (approx. 1 μg protein per binding experiment), or an equivalent amount of the control resin (free MBP), was washed with 3 × 100 μL of Binding buffer (20 mM MES, pH 5.5, 0.05% Tween 20) and incubated with *Nt*Phyt (approx. 200 ng) in a total volume of 20 μL of the same buffer for 1.5 h at 4 °C with shaking. After centrifugation at 2300× *g* for 5 min, unbound *Nt*Phyt was eliminated using 2 × 200 μL washes with Binding buffer, and the resin-bound proteins were eluted using 5 min incubations at room temperature with 3 × 25 μL of 50 mM MES buffer, pH 5.5, containing 500 mM NaCl and 20 mM maltose. Equal aliquots of the combined eluates were used to determine *Nt*Phyt activity (see below). In parallel, aliquots of eluates were analyzed using Western blotting with HisProbe-HRP detection (to evaluate the amount of *Nt*Phyt-His in the samples) and SDS-gel electrophoresis with Coomassie staining (to evaluate the amount of MBP and MBP-Tubic fusions). Experiments with *Nt*Phyt binding were performed in triplicate. Data for the *Nt*Phyt proteolytic activity are presented as means from these experiments.

### 4.7. Confocal Fluorescence Microscopy

Microscopic visualizations were performed using Nikon C2plus (Nikon, Tokyo, Japan) and Zeiss LSM900 Airyscan (Carl Zeiss, Oberkochen, Germany) confocal microscopes equipped with 486 and 562 nm and 488 and 590 nm laser pairs for excitation GFP and RFP, respectively. Images were acquired separately to minimize channel cross-talk. Images were acquired using CFI Plan Fluor 10×/0.3 and Plan-Apo VC 60×/1.2 water-immersion lens (Nikon) and Plan-Apochromat 20×/0.8 and Plan-Apochromat 63×/1.40 Oil DIC oil-immersion lens (Zeiss). Data were reproducible over at least three independent experiments. Confocal images were processed in NIS Elements Viewer (AR 5.42.06, Nikon, Japan) and Zen Microscopy Software (3.4.91.00000, Carl Zeiss, Germany), respectively, and arranged and annotated in Photoshop (Adobe Inc., San Jose, CA, USA)

### 4.8. Phytaspase Activity Determination

Proteolytic activity of phytaspase in total *N. benthamiana* leaf extracts and in eluates from the affinity resin was determined using the Ac-VEID-AFC [AFC, 7-amino-4-(trifluoromethyl) coumarin] fluorogenic peptide substrate (California Peptide Research, Salt Lake City, UT, USA) as described in [46]. Peptide substrate was used at a final concentration of 20 μM. Protein samples were 2- or 10-fold diluted (for the endogenous and the recombinant enzymes, respectively) before activity measurements. Kinetic measurements of relative fluorescence increase were performed in B1 buffer, pH 5.5, containing 0.5 M NaCl at 30 °C. The FLUOstar OPTIMA reader (BMG Labtech, Ortenberg, Germany) equipped with 405 nm excitation and 520 nm emission filters and the Fluoroskan Ascent reader (Thermo Fisher Scientific, Waltham, MA, USA) equipped with 390 nm excitation and 510 emission filters were used to quantitate fluorescence intensities. Data are presented as means ± SD from three independent experiments. Statistical significance was analyzed using a *t* test with Benjamini-Hochberg correction. *p* < 0.05 were considered significant.

### 4.9. Virus-Induced Gene Silencing

*Agrobacterium* C58C1 cultures containing TRV1 and TRV2:NbTLP1 or TRV2:NbTLP1&14 were mixed at a 1:1 ratio and infiltrated into *N. benthamiana* leaves at the four-leaf stage. TRV1 with an empty TRV2 vector (TRV:00) and TRV1 with the TRV2 plasmid containing an insert for phytoene desaturase (PDS) silencing (TRV2:PDS) served as controls. Typically, one month after starting viral infection, total RNA samples were obtained from all types of plants using the RNeasy Plant Mini Kit (Qiagen, Hilden, Germany), reverse transcribed using RevertAid reverse transcriptase (Thermo Scientific, Waltham, MA, USA), and used to determine relative levels of NbTLP1 and NbTLP14 mRNAs. Real time qPCR reactions were performed with the qPCRmix-HS SYBR (Evrogen, Moscow, Russia) and NbTub1_qPCR_dir, NbTub14_qPCR_dir, and NbTub_qPCR_rev primers using the C1000 Thermal Cycler equipped with the CFX96 Real Time System (Bio-Rad Laboratories, Hercules, CA, USA). The 2^−∆∆Ct^ method was used to calculate the relative changes in gene expression using 18S rRNA as a reference. Leaves of the silenced plants were then infiltrated with agrobacteria bearing the protein-encoding constructs (*Nt*Phyt-mRFP and LTI6b-EGFP). At 2 dpi, leaves were treated with 20 µM antimycin A overnight in the dark and inspected using confocal fluorescence microscopy.

### 4.10. Western Blot Analysis

After the addition of 5xSample Buffer (250 mM Tris-HCl, pH 6.8, 5 mM EDTA, 10% SDS, 30% glycerol, 50 mM DTT), samples were boiled for 10 min and fractionated using SDS-polyacrylamide gel electrophoresis (10% and 12% gels and 6–12% gradient gels were used) [47]. Separated proteins were electrophoretically transferred onto a polyvinylidene fluoride (PVDF) membrane. For the Western blot visualization of *Nt*Phyt-His, HisProbe-HRP (horseradish peroxidase) conjugate (Thermo Scientific, Waltham, MA, USA) was used. Detection of the EGFP- and mRFP-containing proteins was performed using monoclonal anti-EGFP 3A9 antibody [48] and rabbit polyclonal anti-tRFP antibodies (Evrogen, Moscow, Russia; at 1:2000 dilution), respectively, in combination with secondary anti-mouse or anti-rabbit IgG linked to HRP (Thermo Scientific, Waltham, MA, USA). Chemiluminescence detection was performed with ECL Western Lightning Plus (PerkinElmer, Shelton, CT, USA) or WesternBright ECL (Advansta, San Jose, CA, USA) reagents using the ChemiDoc Imaging System (Bio-Rad Laboratories, Hercules, CA, USA).

## Data Availability

Data are contained within the article and Supplementary Materials.

## Supplementary Materials

The following supporting information can be downloaded at: https://www.mdpi.com/article/10.3390/ijms26052236/s1.
