# Supplementary material for: Retrograde Transport of Tobacco Phytaspase Is Mediated by Its Partner, Tubby-like F-Box Protein 8
Source: Int J Mol Sci. 2025 Mar 2;26(5):2236. doi: 10.3390/ijms26052236 (PMC11900523; doi:10.3390/ijms26052236)
Supplement: Supplementary file 1 [file ijms-26-02236-s001.zip › ijms-3370572-supplementary.pdf]

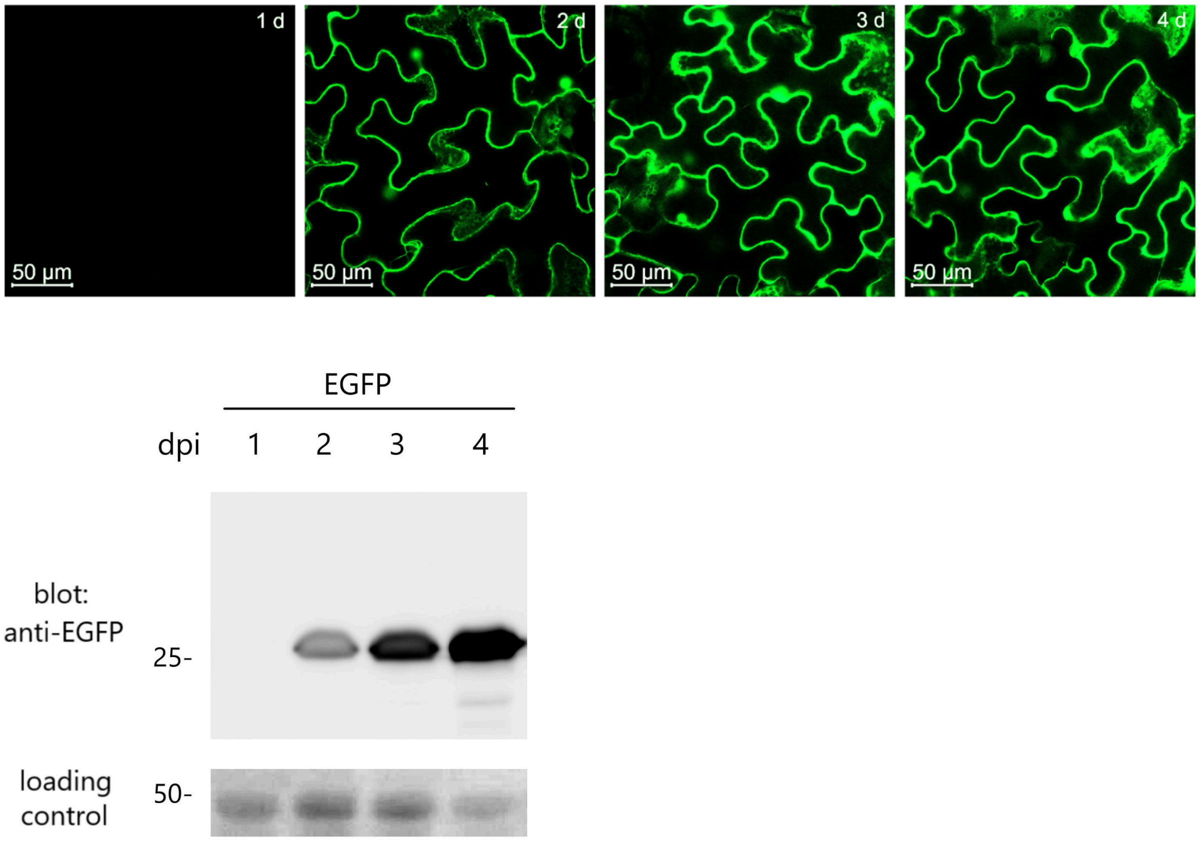

Supplementary Figure S1.

Gradual accumulation of free EGFP in *N. benthamiana* cells upon leaf infiltration with agrobacteria bearing the pLEX_EGFP plasmid. The samples were analyzed by fluorescence microscopy (the upper row) and Western blotting (the bottom row) at 1 to 4 dpi.

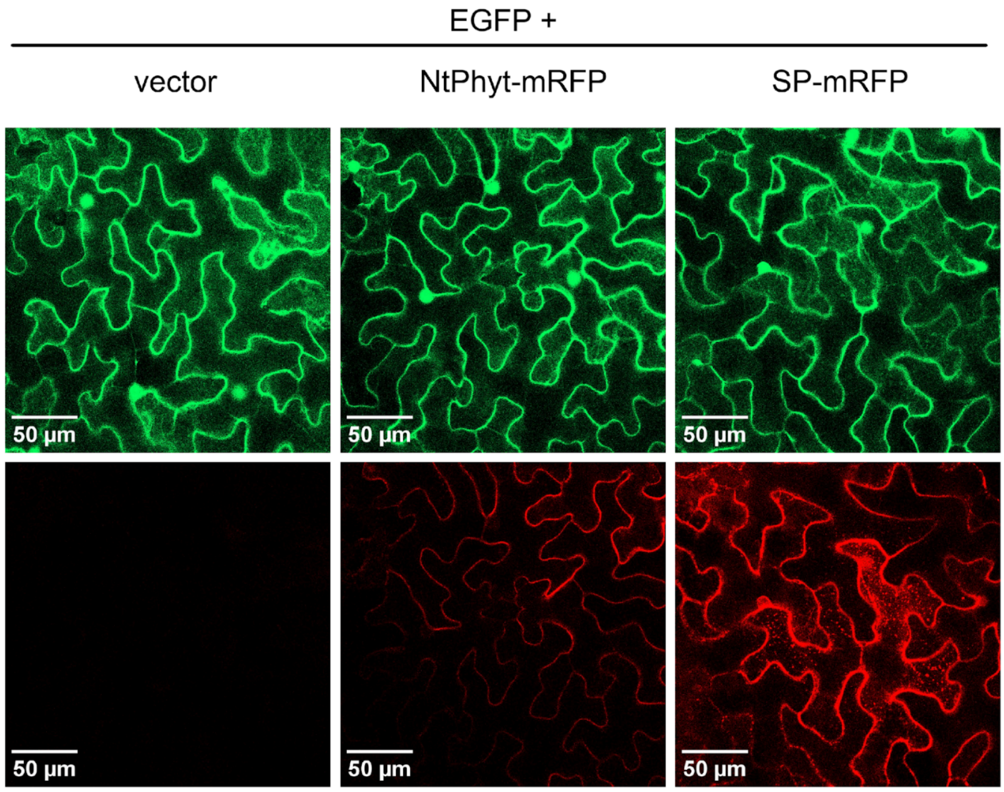

Supplementary Figure S2.

*Nt*Phyt does not interfere with free EGFP production/fluorescence in *N. benthamiana* cells. Leaves of *N. benthamiana* were co-infiltrated with agrobacteria bearing the EGFP-encoding plasmid and the *Nt*Phyt-mRFP- or SP-mRFP-encoding plasmid, or the empty vector. Fluorescence microscopy images (the upper row, green channel; the lower row, red channel) were taken at 2 dpi. Note that the SP-mRFP signal is expectedly much stronger than that of *Nt*Phyt-mRFP.

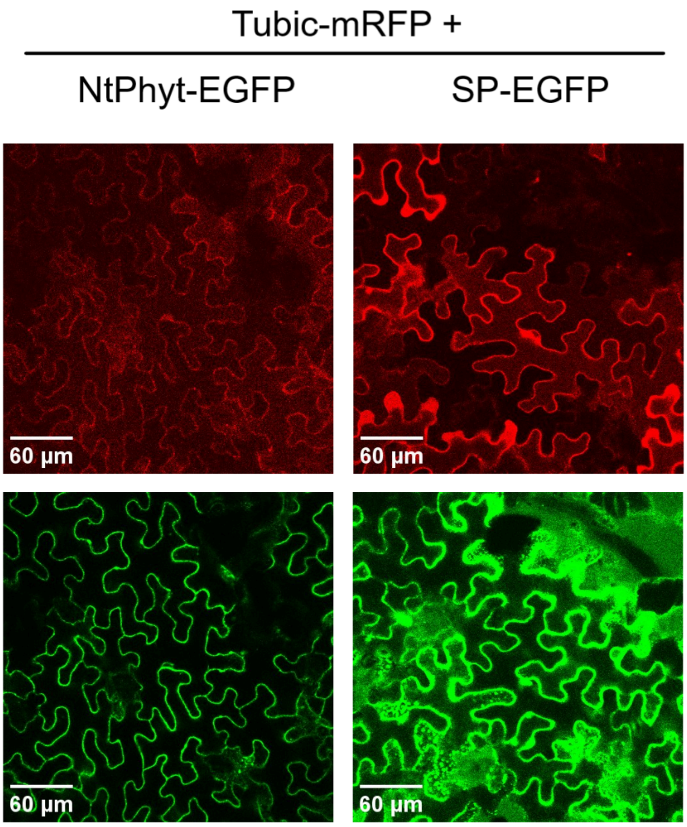

Supplementary Figure S3.

Quenching of the Tubic-mRFP fluorescence signal upon co-production of *Nt*Phyt-EGFP, but not of SP-EGFP, in *N. benthamiana* leaf cells. Images were taken at 2 dpi.

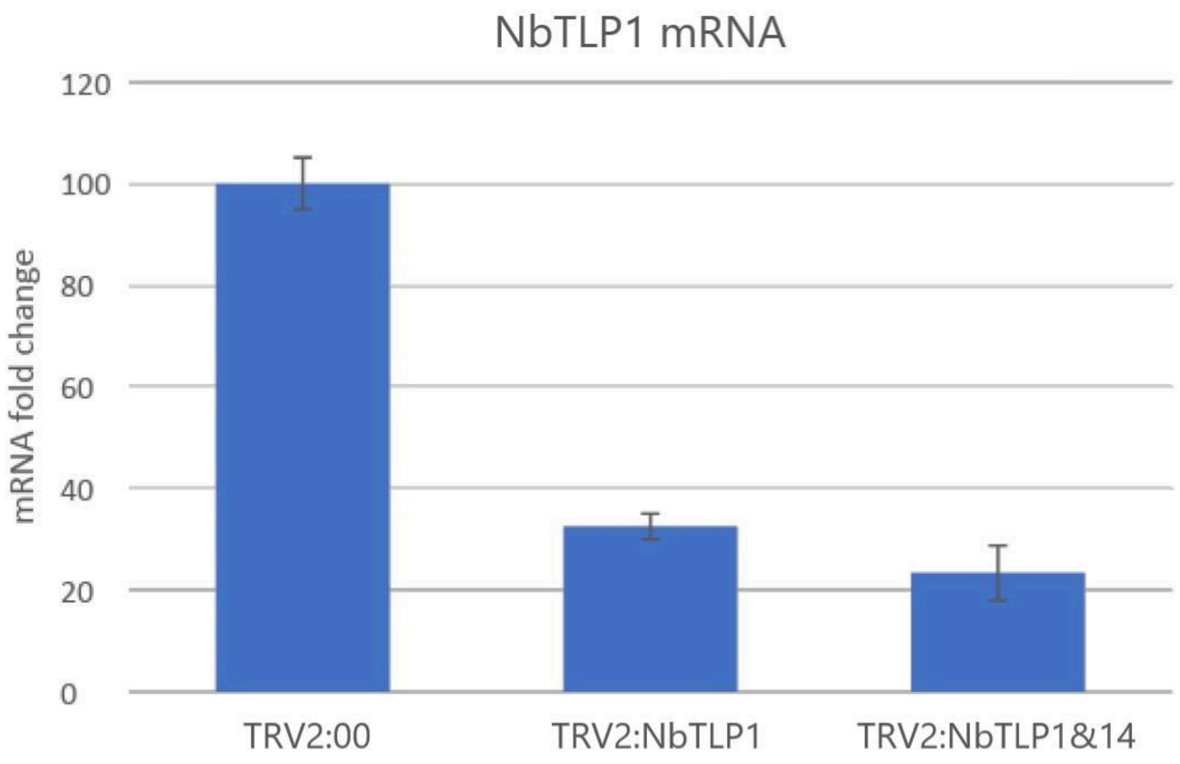

Supplementary Figure S4.

Down-regulation of the NbTLP1 mRNA levels in *N. benthamiana* leaves achieved with TRV2:NbTLP1 and TRV2:NbTLP1&14 constructs, relatively to the empty TRV2 vector (TRV2:00). Relative changes in gene expression were calculated using 18S rRNA as a reference.

Supplementary Table S1.

List of the primers used in this study.

#	Primer name	Sequence
1	Tubic_Bam_dir	CAC GGA TCC ATG TCT TTC CGC AGT ATC GCT C
2	Tubic_Sac_Xho_rev	CTC CTC GAG CTC CTA TTC ACA TGC CAA CTT TGT GTC
3	F-box_z_Sac_rev	CTC GAG CTC CTA CTT AAG AGA GAC CGG AAA G
4	Tub_dom_Bam_dir	CAC GGA TCC CAG CCT GGT TTT CGA GAT G
5	pET28_Sph_dir	GAA TGG TGC ATG CAA GGA G
6	MBP_z_Bam_rev	CAC GGA TCC TTA AGT CTG CGC GTC TTT C
7	NtTubic_Nco_dir	CAA CCA TGG CCT CTT TCC GCA GTA TCG CTC G
8	NtTubic_Bam_rev	GTG GAT CCT GTG CTT CAC ATG CCA ACT TTG TG
9	NtTubic_LP/AA_ rev	CGA AGT AAC TCT GGT GCA GCG CTT GCC CAA CAA CTG
10	NbTub1_VIGS_Kpn_dir	TTG GTA CCG TCA TGC ACT CGG TCC CTG C
11	NbTub1_VIGS_Bam_rev	ATG GAT CCG AGC GCT GCC AAA TTC TGT TG
12	NbTub1&14_VIGS_Xho_dir	TTC TCG AGC TCG TAA ACA TGT TGT TGC
13	NbTub1&14_VIGS_Mlu_rev	AAT ACG CGT AAA TTT GAC CTC AGT TTT CC
14	NbTub1_qPCR_dir	AGT TCA TAA TTT ATG ATA CG
15	NbTub14_qPCR_dir	AGT TCA TAA TCT ATG ATA CC
16	NbTub_qPCR_rev	CAT GTG CAA TTT TGT AGC
